# Bioactive Compounds from *Posidonia oceanica* (L.) Delile Impair Malignant Cell Migration through Autophagy Modulation

**DOI:** 10.3390/md16040137

**Published:** 2018-04-21

**Authors:** Manuela Leri, Matteo Ramazzotti, Marzia Vasarri, Sara Peri, Emanuela Barletta, Carlo Pretti, Donatella Degl’Innocenti

**Affiliations:** 1 Dipartimento di Scienze Biomediche, Sperimentali e Cliniche “Mario Serio”, Università degli Studi di Firenze, viale Morgagni 50, 50134 Firenze, Italy; manuela.leri@unifi.it (M.L.); matteo.ramazzotti@unifi.it (M.R.); marzia.vasarri@unifi.it (M.V.); sara.peri@student.unisi.it (S.P.); emanuela.barletta@unifi.it (E.B.); 2 Dipartimento di Neuroscienze, Psicologia, Area del Farmaco e Salute del Bambino (NEUROFARBA), Università degli Studi di Firenze, viale Pieraccini 6, 50139 Firenze, Italy; 3 Dipartimento di Scienze Veterinarie, Università degli Studi di Pisa, viale delle Piagge 2, 56124 Pisa, Italy; carlo.pretti@unipi.it; 4 Centro Interuniversitario di Biologia Marina ed Ecologia Applicata “G. Bacci”, Viale N. Sauro, 4, 57128 Livorno, Italy

**Keywords:** *Posidonia oceanica*, autophagy, cell migration, gelatinase, HT1080 cell line

## Abstract

*Posidonia oceanica* (L.) Delile is a marine plant with interesting biological properties potentially ascribed to the synergistic combination of bioactive compounds. Our previously described extract, obtained from the leaves of *P. oceanica*, showed the ability to impair HT1080 cell migration by targeting both expression and activity of gelatinases. Commonly, the lack of knowledge about the mechanism of action of phytocomplexes may be an obstacle regarding their therapeutic use and development. The aim of this study was to gain insight into the molecular signaling through which such bioactive compounds impact on malignant cell migration and gelatinolytic activity. The increase in autophagic vacuoles detected by confocal microscopy suggested an enhancement of autophagy in a time and dose dependent manner. This autophagy activation was further confirmed by monitoring pivotal markers of autophagy signaling as well as by evidencing an increase in IGF-1R accumulation on cell membranes. Taken together, our results confirm that the *P. oceanica* phytocomplex is a promising reservoir of potent and cell safe molecules able to defend against malignancies and other diseases in which gelatinases play a major role in progression. In conclusion, the attractive properties of this phytocomplex may be of industrial interest in regard to the development of novel health-promoting and pharmacological products for the treatment or prevention of several diseases.

## 1. Introduction

The angiosperm *Posidonia (P.) oceanica* (L.) Delile is a sea-grass widely distributed in the Mediterranean Sea forming dense underwater meadows that cover tens to thousands of square kilometres. In the sea coast of Western Anatolia, the decoction of *P. oceanica* leaves became an herbal preparation used for diabetes mellitus and hypertension remedies. In fact, the antidiabetic and vasoprotective properties of a *P. oceanica* extract have been confirmed in treated alloxan diabetic rats [1]. Further data on the bioactivity of *P. oceanica* have been described, such as antibacterial and antifungal properties as well as antimelanogenic and lypolitic activities [2] of a crude extract of leaves.

Hence, the interest in *P. oceanica* as a potential source of novel natural products that are useful for the treatment or prevention of different pathological processes has greatly increased. Consequently, the molecular mechanisms through which the bioactive compounds of *P. oceanica* exert their activities need to be clarified.

**Figure 1:**
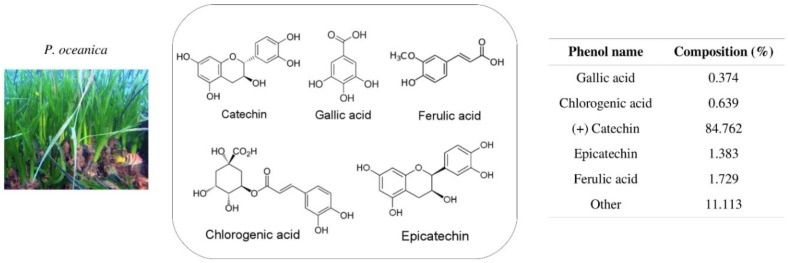
Polyphenolic profile characterization of *P. oceanica* extract (POE) by UPLC analysis [3].

In our previous work, we used UPLC analysis to characterize the polyphenolic profile of a hydrophilic fraction of *P. oceanica* extract (POE), evidencing a large amount of catechins and minor amounts of polyphenols [3] (Figure 1). Very low doses of POE showed the ability to drastically reduce the motility of the highly invasive HT1080 fibrosarcoma cell line. This effect was due to the concomitant presence of phenolic compounds in the total extract that synergistically decreased the expression of gelatinases and directly inhibited gelatinolytic activity [3].

Gelatinases, as members of the matrix metalloproteinase MMP family, are fundamental players in maintaining the cellular environments needed by several physiological processes. However, they could participate in the development of important physio-pathological chronic processes, such as neurodegeneration, inflammation and cancer development. Specifically, in cancers, MMPs take part in extracellular matrix degradation and cancer cell invasion and metastasis making cancer cells able to migrate and propagate [4].

Recent studies have correlated cancer cell migration with autophagy (i.e., macroautophagy), the major cellular digestion process conserved from yeast to mammals [5]. Autophagy process leads cells to digest parts and components of their own cytoplasm to overcome intracellular or environmental stress conditions, as nutrient deprivation or hypoxia [6,7].

Generally, motile and invasive cancer cells require autophagy augmentation to survive in stressful conditions during invasive and metastatic processes, but recently it has been proven that autophagy could contrast with early stages of the epithelial to mesenchymal transition (EMT) in which cancer cells lose typical epithelial phenotype properties and acquire motility features [8]. The literature reports that nutrient deprivation in glioblastoma cells impairs both migration and invasion and reverts EMT [9]. On the contrary, the knockdown of key autophagy inhibitors has been proven to stimulate cell migration and β-integrin recycling in HeLa cells [10].

Many different signaling pathways have been described to modulate autophagy, specifically by influencing the activity of the mammalian target of rapamycin (mTOR), its key master regulator [11].

Phosphatidylinositol-3-kinases (PI3K) /Protein Kinase B (AKT)/mTOR and Ras/Raf/Mitogen-activated protein kinase/ERK kinase (MEK)/extracellular-signal-regulated kinase (ERK) (Ras/MEK/ERK) signal transduction pathways are well-established upstream regulators of mTOR, the master autophagy suppressor. They are the main cellular mechanisms for controlling cell proliferation, survival, differentiation, metabolism and motility and both are well-known mediators of autophagy in response to extracellular stimuli [12]. AKT positively modulates mTOR activity and, accordingly, inhibition of AKT could stimulate the autophagy process by lowering mTOR activity [13] and consequently enhancing Beclin-1 levels, one of the mTOR downstream targets. Beclin-1 is a well-established marker of the early stages of autophagy [14] that promotes nucleation of the autophagic vesicle named the autophagosome. During autophagosome maturation, the phosphatidylethanolamine lipidation of the protein LC3-I to LC3-II occurs. Unlike Beclin-1, LC3-II is a marker of autophagosome full maturation that, in turn, forms the autophagolysosome upon fusion with a lysosome. Lysosomal hydrolases then act to break down the cargo mainly through p62 (i.e., sequestosome 1, SQTSM1), a ubiquitin-binding scaffold protein that drives attached protein targets to autophagosomes for selective degradation (the so-called selective autophagy) and is specifically considered a marker of the degradation phase of autophagy [15].

Beyond its known role in cell survival, the PI3K/AKT axis promotes cell migration in several cancer cell lines, including the HT1080 cell line [16] by increasing cell motility and expression and/or the activity of gelatinases [17]. Specifically, cell-surface insulin-like growth factor-1 receptor (IGF-1R), which senses IGF-1 levels, is an upstream regulator of PI3K/AKT through which it enhances cell proliferation and migration [18]. Pieces of evidence have indicated that the activated IGF-1R translocates to the nuclei of several human cancer cells and regulates, by acting as a transcription regulator, cancer cell migration through the modulation of MMP-2 expression [19,20]. Thus, suppression of the IGF-1R/PI3K/AKT/mTOR signaling pathway could interfere with IGF-1R or AKT activity and could impair cell migration by affecting the expression of gelatinases [21]. Furthermore, it has been demonstrated that the dietary flavonoid, luteolin, reduces the migration of HT1080 cells and attenuates the EMT process via suppression of the IGF-1R/PI3K/AKT/mTOR pathway [22].

As expected from a very basic cell process with a fundamental role in cell homeostasis, an intricate interplay between autophagy and many other processes has been described. It is therefore useful to refer to updated guidelines for the use and interpretation of assays for monitoring autophagy [5].

In order to better clarify our previous findings on POE effects, in this study we aimed to investigate the role of autophagy in the inhibition of the motility of HT1080 cells. In this framework, we used wound-healing assay, zymography, Western blotting as well as confocal microscopy to investigate whether POE could exert, in the absence of other stimuli, its inhibitory effects on migration by modulating autophagy without affecting cell viability.

## 2. Results and Discussion

### 2.1. Biochemical Characterization of POE

Our previous results reported that the water–ethanol extraction method is able to efficiently recover polyphenols and carbohydrates from minced *P. oceanica* dried leaves [3]. In the present study, POE was shown to contain 13 ± 2 mg/mL glucose equivalents of carbohydrates and 5.7 ± 0.3 mg/mL gallic acid equivalents of polyphenols. Their bioactive antioxidant properties were further investigated, evidencing radical scavenging and antioxidant activity of 12.8 ± 0.7 mg/mL and to 1.5 ± 0.3 mg/mL ascorbic acid equivalents, respectively. These values, reported in Table 1, although slightly lower, are in agreement with our previous report, confirming the robustness of our extraction procedure [3]. All treatments hereafter described were done with 1:500 or 1:1000 dilutions of freshly prepared POE corresponding to polyphenol concentrations of 11.4 and 5.7 μg/mL gallic acid equivalents. (corresponding to 67 μM and 33.5 μM), respectively.

### 2.2. HT1080 Cell Migration in Heat-Inactivated Fetal Bovine Serum (FBS) Medium

**Figure 2:**
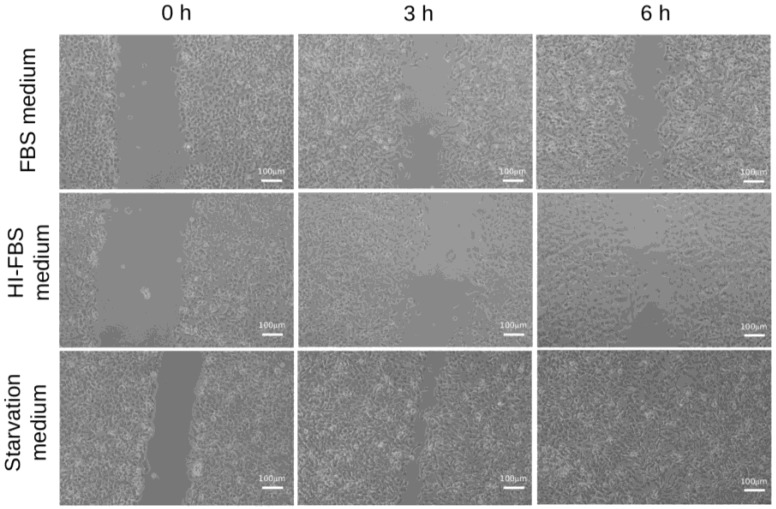
Wound-healing assays of HT1080 cells growing in FBS medium (**top**), HI-FBS (**middle**) or starvation medium (**bottom**). Three time points are shown. HT1080 cells maintained their motility phenotype in HI-FBS medium, a favourable condition that is not associated with a basal increase in autophagy.

Classically, starvation medium condition is used to induce EMT and activate the motility features of cells [23]. Abundant literature reports that heat inactivation of FBS (HI-FBS) markedly decreases the levels of several plasma factors, such as MMPs, chemokines and cytokines, compared to the condition with FBS [24]. Hence the use of HI-FBS medium decreases unwanted and unpredictable serum factors that could interfere with various cellular processes. In our experimental set-up, the incubation of cells in conditions such as starvation, FBS medium or HI-FBS medium allowed us to verify the innate motility phenotype of HT1080 cells in correlation with growth conditions. By means of a wound-healing assay, we found that the motility of HT1080 cells decreased but was not abolished in both serum-containing conditions with respect to the starvation medium (Figure 2). In addition, the invasive potential of cells was found to be very similar between HI-FBS and FBS media, clearly showing that the innate motility features of HT1080 cells are maintained in non-stressful, serum-containing conditions. Accordingly, all further experiments were based on cells grown in HI-FBS medium, so that unwanted side effects on cellular processes were minimized while the typical motility phenotype of HT1080 cells was maintained.

### 2.3. HT1080 Cell Migration Impairment Following POE Treatment

**Figure 3:**
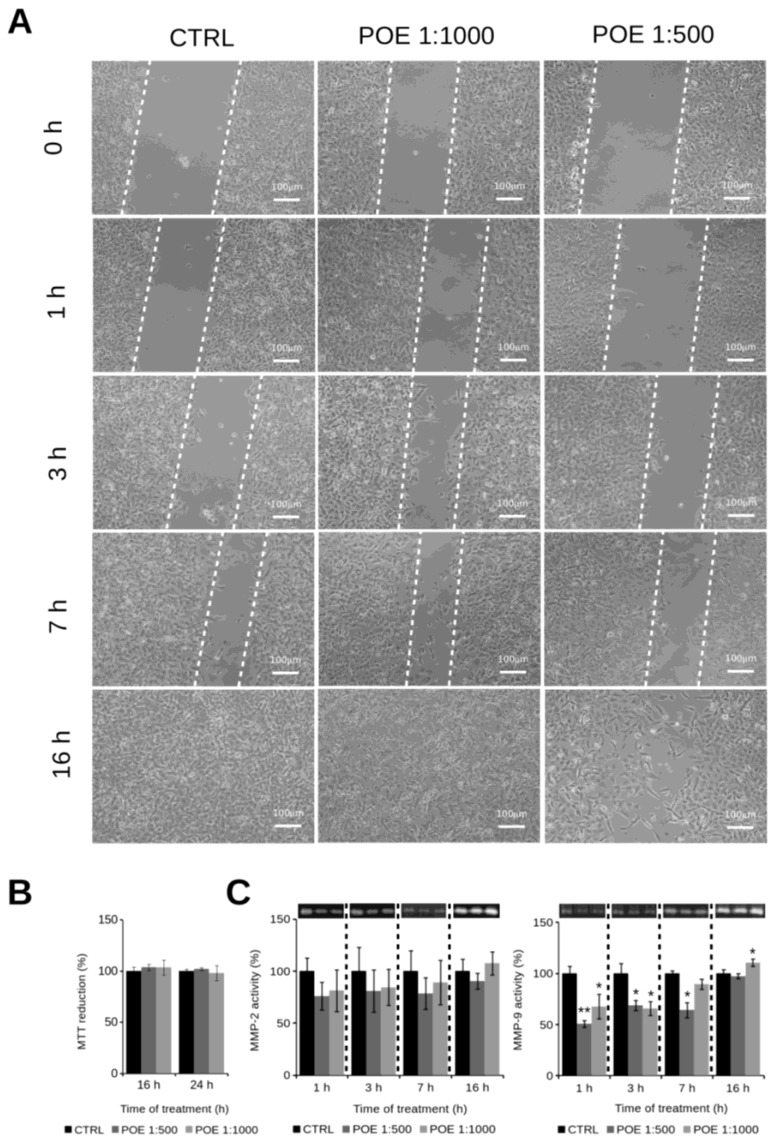
(**A**) Wound-healing assays of HT1080 cells growing in HI-FBS medium, treated or not treated with 1:1000 and 1:500 POE dilutions. The dashed lines mark the boundaries of the wound area; (**B**) MTT assay showing a substantial absence of cell toxicity by POE; (**C**) Gelatin zymography of HT1080 conditioned medium collected at 1, 3, 7 and 16 h time points of HT1080 cells cultured in the presence or absence of 1:500 and 1:1000 POE dilutions. The ability of HT1080 cells to migrate to the cell-free space is drastically reduced by the addition of POE in the absence of cell toxicity, in a dose- and time-dependent manner influencing the production or the release of both MMP-2 and MMP-9. *: *p*-value < 0.05; **: *p*-value < 0.01; Student *t*-test, *n* = 3.

Having established that the motility phenotype of HT1080 cells was maintained in HI-FBS medium, we performed the wound-healing assays in the presence of 1:500 and 1:1000 POE dilutions. Both treatments did not affect cell viability (Figure 3B) and reduced cell motility in the first seven hours after treatment, an effect that was not present after 16 h (Figure 3A). Such results were further supported by gelatin zymography aimed at monitoring the activity of MMP-2 and MMP-9, well-known markers of cell migration, in conditioned media collected at different time points. The zymography analysis showed a total gelatinase activity reduction after 1:500 and 1:1000 POE treatments. In particular, the 1:500 and 1:1000 treatments led to an observed MMP-2 activity decrease of about 22% during the first 7 h. A more pronounced behaviour during the same time range was found for MMP-9 activity, with a reduction of about 35% for the first 7 h. Such trends were not observed in non-treated control cells at the same times. Gelatinase activities were clearly recovered after 16 h POE treatments, at any tested dose (Figure 3C). These results confirmed that the anti-invasive properties of bioactive compounds from POE on the highly motile HT1080 cell line were, at least in part, due to a transient reduction of gelatinase production or secretion.

### 2.4. POE Treatment Induces Autophagy in HT1080 Cells

**Figure 4:**
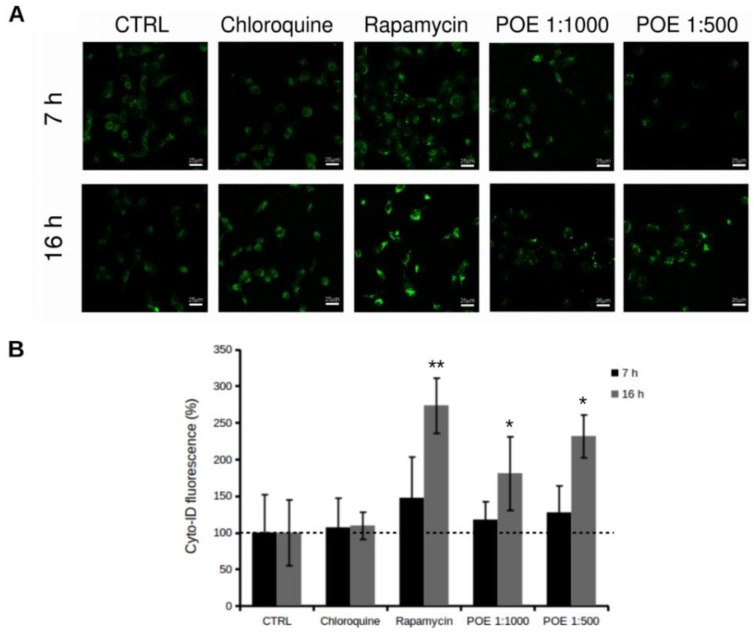
Increase of autophagy in HT1080 cells following POE addition. (**A**) Autophagy specific dye Cyto-ID^®^ was analysed by confocal microscopy in HT1080 cells after 7 h and 16 h of treatment with 1:500 and 1:1000 POE dilutions. Rapamycin and chloroquine were used as positive and negative controls, respectively. Autophagy vacuoles increased upon addition of POE in a dose- and time-dependent manner compared to untreated control cells (CTRL); (**B**) Quantification of Cyto-ID^®^ fluorescence. Autophagy significantly increased upon POE treatment in a dose- and time-dependent manner. *: *p*-value < 0.05; **: *p*-value < 0.01; Student *t*-test, *n* = 3.

In order to examine the molecular mechanism(s) through which compounds of POE exert the reduction of cell motility demonstrated above, we initially investigated several features and markers of the autophagy process. In fact, recent studies have reported that cell migration could be modulated by autophagy [8]. Firstly, we analysed HT1080 cells treated with POE using Cyto-ID^®^ staining, a selective fluorescent marker for autophagic vacuoles [25]. Immunofluorescence results showed a clear increase in autophagy in HT1080 treated cells compared to untreated control cells (Figure 4). The increase of fluorescence intensity and the number of labelled particles in treated cells suggested that POE treatment promotes the formation of autophagosomes in the cytosol. Specifically, we observed a significant increase in Cyto-ID^®^ intensity of about 80% and 130% at 1:1000 and 1:500 POE dilutions after 16 h treatment, respectively, compared to untreated cells (Figure 4B). The autophagy modulation was confirmed by using chloroquine and rapamycin as controls. In particular, cells treated with chloroquine did not show an increase in Cyto-ID^®^ signal intensity, confirming its autophagy inhibitory role [26]. On the contrary, the autophagy inducer, rapamycin [27], significantly increased the Cyto-ID^®^ signal after 16 h of treatment (Figure 4B).

**Figure 5:**
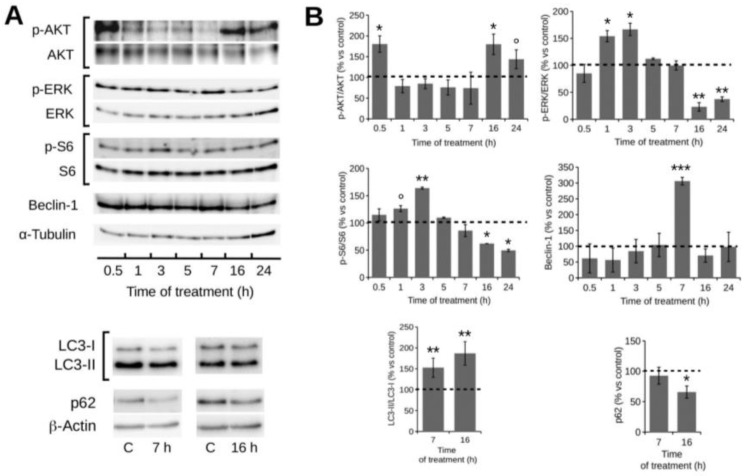
Western blotting analysis of the status of autophagy markers in HT1080 cells following 1:500 POE treatment. (**A**) Representative Western blots of all of the assayed markers; (**B**) Quantification of signals from a densitometry analysis of at least three independent experiments. Error bars represent standard errors. POE treatment reduces AKT and S6 phosphorylation and increases ERK phosphorylation at the early stages of the HT1080 cell response, while, at late stages, an inverse trend can be observed for AKT and ERK. Beclin-1 progressively increases, reaching the highest peak at 7 h. The LC3-II form increases only at the late stages of the response with a concomitant decrease in p62. °: *p*-value < 0.1; *: *p*-value < 0.05; **: *p*-value < 0.01; ***: *p*-value < 0.001; Wilcoxon test, *n* = 3.

Given this enabling evidence, we investigated the most common signaling pathways controlling autophagy by Western blotting, focusing our analysis on the upstream and downstream pathways of mTOR, the most well-known suppressive regulator of autophagy. As shown in Figure 5, we assayed signaling pathways in HT1080 cell lysates at different times during 1:500 POE treatment, from 0.5 h to 24 h (considering the ability of the chosen dose to consistently activate autophagy in the absence of toxicity, as shown in Figure 4 and Figure 3B). We showed a reduction in the phosphorylation levels of AKT at 1 h (20 ± 16%), 3 h (15 ± 12%) and 5 h (24 ± 18%) during POE treatments. Since AKT activation by phosphorylation is considered a pro-survival stimulus related to the PI3K/AKT/mTOR survival pathway, the reduction we observed could be potentially ascribed to a pro-apoptotic stimulus. We further monitored the phosphorylation status of ERK, showing an increase in the phosphorylation levels of ERK at 1 h and 3 h, of 54 ± 10% and 66 ± 11%, respectively. The concomitant and inverse correlations between the phosphorylation status’ of AKT and ERK is common in drug-induced stimulation of autophagy [28,29,30], and it is probably one of the several feedback mechanisms involving pathways fundamental for cellular homeostasis. After 7 h of treatment, the effect of the AKT activity reduction led to a decrease in S6 phosphorylation (14 ± 11%), one of the main targets used to monitor mTOR activity as a mainstream inhibitor of autophagy. At this time point, AKT maintained a low level of phosphorylation (26 ± 38%) while ERK phosphorylation returned to control levels. At the 16 h time point, AKT recovered its baseline phosphorylation status while both ERK and S6 phosphorylation continued to decline, as evidenced after 24 h of treatment. We further investigated the dynamics of Beclin-1 levels, since this alteration is considered a crucial event for the initial step of autophagosome formation (so, a downstream event in the autophagic signaling cascade) and therefore, a marker of effective activation of the autophagy process. We observed a progressive increase from 0.5 h up to a peak of activation after 7 h of treatment (206 ± 12%) with Beclin-1, supporting the observed autophagy enhancement. At the 7 h time point, we further showed initial traces of autophagosome maturation, as supported by a slight increase in the conversion of LC3-I to LC3-II (the lipidated form) (86 ± 8%), a well-accepted marker of lengthening of the autophagosome. The level of p62 protein, a marker of the degradation phase of autophagy, proved to be substantially unchanged with respect to the untreated control at this 7 h time point, suggesting that the autophagolysosome formation was in its early stage. At the 16 h time point, we detected clear signs of full autophagolysosome formation, with a net reduction in Beclin-1 (that remained unchanged until 24 h, the latest time point we measured), a marked rise in the levels of LC3-II lipidation and a net reduction inp62 protein levels (35 ± 3%). It is important to underline that the kinetics of the autophagy process activation upon POE treatment in all of the above-mentioned markers is in agreement with the results obtained with the Cyto-ID^®^ analysis. In fact, the highest level of Beclin-1 was established at 7 h and correlated well with the initial autophagosome formation that was already evident at 7 h in the Cyto-ID^®^ results. The concomitant decrease in Beclin-1, the marked presence of the LC3-II lipidated form and the fall in the p62 protein level after 16 h of POE treatment suggested that the autophagy process was fully mature. This further supports the evidence for the effective maturation of autophagolysosomes, reinforcing the results obtained with Cyto-ID^®^ (Figure 4), which in fact is reported to co-localize with LC3 [31].

### 2.5. Autophagy Modulation by POE Decreases Cell Migration 

**Figure 6:**
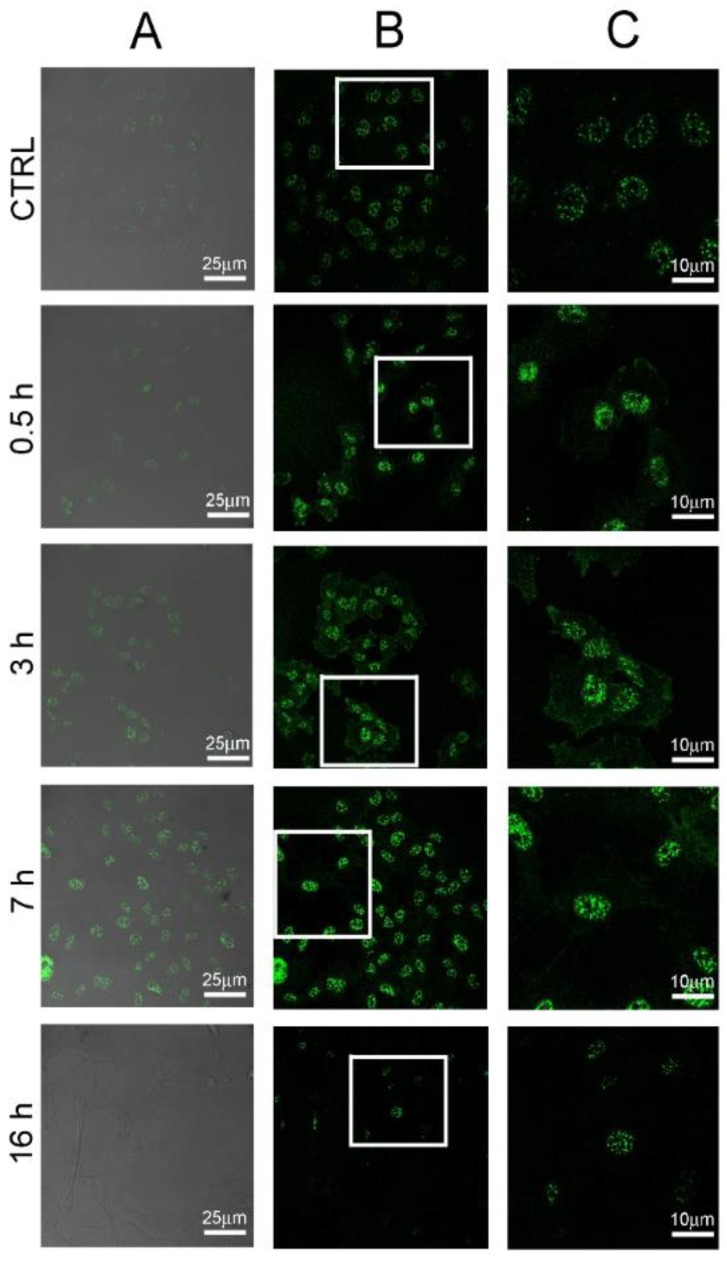
Immunofluorescence analysis of cell-surface insulin-like growth factor-1 receptor (IGF-1R) localization. HT1080 cells untreated (CTRL) or treated with 1:500 POE for 0.5 h, 3 h, 7 h and 16 h. (**A**) Merging of differential interference contrast (DIC) and IGF-1R staining with Alexa 488 (green) channels; (**B**) Alexa 488 (green) channel; (**C**) Magnification of selected areas of panel B, showing details of IGF-1R localization within cells.

Observed autophagy modulation by POE treatment could contribute to the HT1080 cell migration reduction that we described above. Recent studies have reported the existence of a correlation between autophagy and cell migration. In particular, AKT phosphorylation and the consequent signaling is described as being positively correlated with cell motility and gelatinase activation. Our results show that POE treatment causes a reduction in motility and a reduction in MMP-2/9 activity, concomitant with the reduction in AKT phosphorylation. The literature further reports that MMP-2 expression, and thus, cell migration, can be regulated by IGF-1R which translocates into the cell nucleus in response to PI3K/AKT signaling [17]. We therefore investigated, by confocal microscopy, the relationships between the cell membrane and nuclear accumulation of IGF-1R in untreated cells or cells treated with POE 1:500. It is important to re-emphasize here that HT1080 cells are widely used as a model of cancer cell migration due to their extremely motile phenotype. Our analysis showed complete accumulation of IGF-1R in the nuclei of untreated cells, confirming its association with motility in this cell line (Figure 6). After 0.5 h of POE treatment, cells showed a marked IGF-1R nuclear localization and consequently, a redistribution on the cell membrane, in particular after 3 h of treatment. The documented early increase in IGF-1R redistribution on the cell surface was found to be reduced at 7 h and fully abolished after 16 h of treatment, confirming that the POE effect was transient and lasted for a few hours only. Our kinetic data regarding IGF-1R accumulation was found to be perfectly coherent with the autophagy signaling data shown above and, more importantly, with the overall change in cell phenotype and behaviour upon POE treatment.

**Figure 7:**
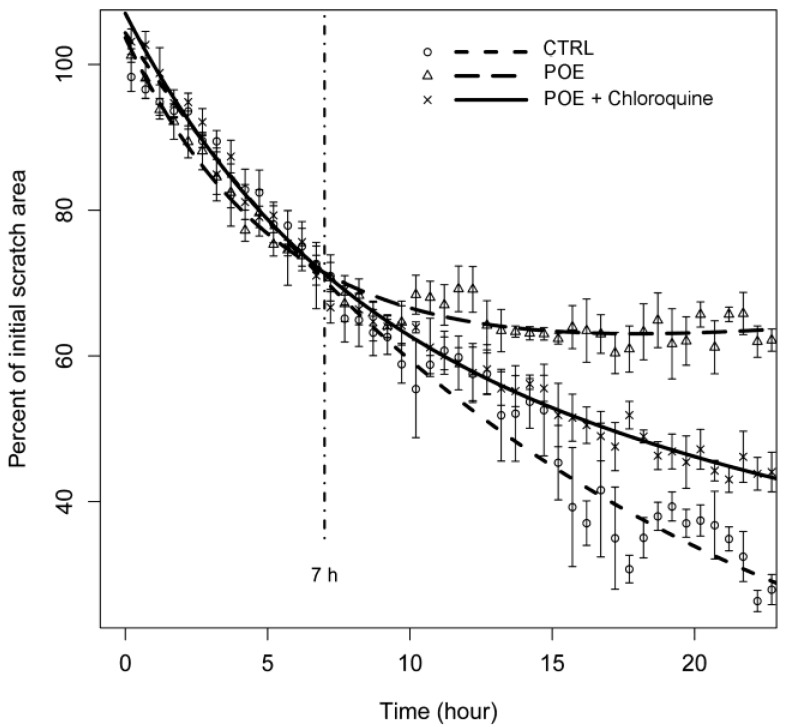
Time-lapse experiment of HT1080 migration following treatment with POE and chloroquine. CTRL: untreated HT1080 cells; POE: HT1080 cells treated with 1:500 POE; POE + Chloroquine: HT1080 cells treated with 10 µM chloroquine after 7 h treatment with 1:500 POE. A clear recovery of motility was induced by the addition of the autophagy inhibitor, chloroquine, indicating the major involvement of autophagy in the migration arrest induced by POE.

We further investigated the relationship between POE-induced autophagy and its effect on cell migration by adding chloroquine, which blocks lysosome acidification, i.e., the last step of the autophagy process. We recorded cell migration in a time-lapse wound-healing assay, and we found that, as depicted in Figure 7, the presence of POE determined a stall in wound area closure kinetics after 7 h, in contrast to what was observed in untreated controls (in agreement with the zymographic data shown in Figure 3). The addition of chloroquine at 7 h in POE treated cells, the most relevant time point for autophagy activation, showed a recovery of the motility phenotype. Taken together, such results demonstrate that the effects of POE on cell migration impairment occur through the modulation of the autophagy process.

## 3. Materials and Methods

### 3.1. Materials

3-(2-Pyridyl)-5,6-diphenyl-1,2,4-triazine-4′,4″-disulfonic acid sodium salt (Ferrozine^®^), α,α-diphenyl-β-picrylhydrazyl (DPPH), Folin–Ciocalteau’s phenol reagent, gallic acid, ascorbic acid, d-glucose, gelatin, Coomassie Brilliant Blue R-250, 1-(4,5-dimethylthiazol-2-yl)-3,5-diphenylformazan (MTT), Dulbecco’s Modified Eagle’s Medium (DMEM), Fetal Bovine Serum (FBS) and Bovine Serum Albumin (BSA) were all purchased from Sigma Aldrich-Merck (St. Louis, MO, USA). 30% Acrylamide/Bis 37.5:1 solution, ammonium persulfate (APS), 1,2-Bis (dimethylamino) ethane (TEMED), Tris/Glycine buffers, nitrocellulose membranes (0.45 μM) blotting membranes and Clarity Western ECL solution were purchased from Bio-Rad. Disposable plastics were from Corning. Photometric measurements from multi-well plates were recorded on an iMARK microplate reader (Bio-Rad, Hercules, CA, USA). When not otherwise specified, all chemicals and solvents, such as ethanol, methanol and n-hexane, were of the highest analytical grade and were purchased from Sigma Aldrich-Merck.

### 3.2. Preparation of P. oceanica Extract

The collection of *P. oceanica* (L.) Delile and the extraction of its hydrophilic components were performed using a previously-described protocol [3]. Briefly, dried *P. oceanica* leaves (collected in July 2016) were minced and suspended overnight in 10 mL of ethanol (70% per gram of leaves) under stirring conditions at 65 °C for 3 h. *P. oceanica* ethanol extract was separated from debris by centrifugation, and the supernatant was mixed in a 1:1 ratio. Hydrophobic compounds were removed by repeatedly shaking, while hydrophilic compounds were mainly contained in the cleaned and recovered phase of the extract and subsequently, were dispensed in aliquots of 1 mL. Batches of the extract were dried by a Univapo™ vacuum-spin concentrator, and then single batches were dissolved in 0.5 mL 70% ethanol in sterile water before use.

### 3.3. Determination of Total Polyphenol Content

The total polyphenol content of POE was determined according to the colorimetric Folin–Ciocalteau method [32]. Scalar volumes of POE were dispensed in a 96-well microplate and diluted with H_2_O (final volume 20 μL). Then, 100 μL of the Folin–Ciocalteu’s phenol reagent (diluted 1:10 in H_2_O) was added to each well. After 5 min of incubation at room temperature (RT), 80 μL of 7.5% sodium carbonate solution was added per well and incubated for further 2 h. The absorbance at 595 nm was recorded with a microplate reader. Polyphenol content was determined by linear regression using gallic acid as a reference in the range of 0–10 μg.

### 3.4. Determination of Total Carbohydrate Content

The total carbohydrate content of POE was determined according to the phenol–sulfuric acid method [33]. Briefly, scalar aliquots of POE were added to a 96-well microplate and diluted with H_2_O (final volume 50 μL), and then 150 μL of concentrated sulfuric acid was added to each well. After 5 min of incubation at RT under continuous shaking, 30 μL of 5% phenol solution was added to each well and heated for 10 min at 90 °C. After cooling to room temperature for 20 min, the absorbance at 490 nm was recorded with a microplate reader. The carbohydrate content was determined by linear regression using d-glucose as a reference in the range of 0–50 μg.

### 3.5. Determination of Radical Scavenging Activity

The radical scavenging activity of POE was determined by adapting the method from Fukumoto and Mazza [34]. Briefly, scalar aliquots of POE were diluted with 95% methanol (final volume 100 μL) and then mixed with 100 μL of freshly prepared DPPH solution (0.15 mg/mL in 95% methanol) in a 96-well microplate. After 30 min of incubation at RT in the dark, the absorbance was recorded at 490 nm with a microplate reader. Radical scavenging activity was determined by linear regression using ascorbic acid as a reference in the range of 0–4 μg.

### 3.6. Determination of Total Antioxidant Activity

The total antioxidant activity of POE was estimated using the FRAP (ferric-reducing/antioxidant power) method [35]. Briefly, scalar aliquots of POE were diluted with water (final volume 50 μL) and 200 μL of Ferrozine™ reagent (10 mM Ferrozine™ in 40 mM HCl:20 mM ferric chloride:0.3 M acetate buffer pH 3.6, ratio 1:1:10) was added to each aliquot in a 96-well microplate. After 5 min of incubation at RT in the dark, the absorbance was measured at 595 nm with a microplate reader. Antioxidant activity was determined by linear regression using 0.1 mg/mL ascorbic acid as a reference in the range of 0–4 μg.

### 3.7. Cell Line and Culture Conditions

The HT1080 human fibrosarcoma cell line was grown in DMEM (Dulbecco’s Modified Eagles Medium) supplemented with 2 mM l-glutamine, 100 μg/mL streptomycin, 100 U/mL penicillin and 10% fetal bovine serum (FBS medium), at 37 °C in a 5% CO_2_-humidified atmosphere. At 90% confluence, cells were detached by trypsinization (trypsin 0.025%-EDTA 0.5 mM) and propagated after appropriate dilution. Medium supplemented with FBS was inactivated at 56 °C for 30 min (HI-FBS medium), and serum-free medium (starvation medium) was used for some of the following experiments. HT1080 cells were grown in the presence or absence of freshly-dissolved POE and appropriate controls.

### 3.8. Cell Viability Assay

Cell viability was assessed using the colorimetric 3-(4,5-dime-thylthiazol-2-yl)-2,5-diphenyltetrazolium bromide (MTT) metabolic activity assay after different cell treatment conditions [36]. In brief, cells were grown in a 24-well plate (5 × 10^5^ cells/well) in HI-FBS medium for 24 h. Then, cells were treated with two different POE dilutions, 1:500 and 1:1000, for 16 h and 24 h, while untreated cells were used as a control. After removing the incubation medium and washing with PBS, 200 mL/well of 0.5 mg/mL MTT solution was added and incubated in the dark at 37 °C for 1 h. Next, after PBS washing, cells were lysed in 200 μL dimethyl sulfoxide (DMSO) and absorbance values were measured at 595 nm with a microplate reader. Data were expressed in terms of percentage with respect to untreated controls.

### 3.9. Cell Migration Assay

Cell migration was assayed using the scratch wound healing assay. HT1080 cells were seeded in 12-well plates at a high density (5 × 10^5^ cells/well) and grown to confluence overnight in three different culture media, namely FBS medium, HI-FBS medium and starvation medium, with or without POE treatment. Next, we made a vertical wound through the cell monolayers using a sterile plastic tip, and plates were washed several times with PBS to remove cell debris and medium. Fresh culture media was added again, and then the cell-free area was observed under phase contrast microscopy, and images were captured at time points ranging from 0 h to 16 h using a Nikon TS-100 microscope equipped with a digital acquisition system (Nikon Digital Sight DS Fi-1, Nikon, Minato-ku, Tokyo). Time-lapse experiments were performed by seeding 1 × 10^6^ cells on 10 cm^2^ culture plates and culturing them in HI-FBS medium supplemented with 20 mM HEPES in order to maintain the desired pH without requiring CO_2_. Cells were treated with and without POE (1:500). After 7 h of incubation with POE, the medium was supplemented with chloroquine (10 µM). The wounded cell-free area was observed under phase contrast microscopy for 24 h at 37 °C. Three frames from the same optical field were captured every 5 min by time-lapse recording, and wound size was analyzed with TScratch software (ETH CSElab, Zurich, Switzerland) and further processed with R statistical software. 

### 3.10. Gelatin Zymography

Gelatinase activity was assayed by gelatin zymography using conditioned medium from HT1080 cell cultures previously seeded at a density of 2 × 10^5^ cells/well in 24-well culture plates and incubated in FBS medium for 18 h. Subsequently, culture medium was removed, and cells were incubated in HI-FBS medium following addition of 1:500 and 1:1000 POE dilutions for up to 16 h, while untreated cells were used as a control. After these incubation time points, culture supernatants were collected and centrifuged at 9700× *g* for 1 min at 4 °C in order to pellet cell debris. Then, 2.5 μL aliquots of conditioned medium from control or POE treated HT1080 cells were electrophoresed under non-reducing conditions in 8% polyacrylamide gels containing 1 mg/mL gelatin. After the electrophoretic separation, gels were washed twice in 2.5% Triton X-100 for 1 h to remove SDS and then incubated at 37 °C for 24 h in reaction buffer (50 mM Tris-HCl pH 7.4, 0.2 M NaCl, 5 mM CaCl_2_, 1 μM ZnCl). Gels were stained with 0.05% Colloidal Coomassie Brillant Blue G-250 dissolved in 1.6% phosphoric acid, 8% ammonium sulfate and 20% methanol and destained in 1% acetic acid. Gelatinase activities appeared as clear bands against a blue background. Zymography images were acquired with a digital scanner.

### 3.11. Analysis of Autophagic Vacuoles

The Cyto-ID^®^ Autophagy Detection Kit (Enzo Life Sciences, Shanghai, China) was used to monitor the induction of autophagy using fluorescence microscopy, in accordance with the manufacturer’s instructions. Cyto-ID^®^ dye selectively labelled the autophagic vacuoles in living cells, so HT1080 cells (5 × 10^4^ cells/well) were seeded for 24 h in a 24-well culture plate containing sterilized glass coverslips. Following treatments for 7 and 16 h with 1:500 and 1:1000 POE dilutions, as well as 0.5 μM of rapamycin and 10 μM of chloroquine as positive and negative controls, respectively, cells were washed twice with PBS and then with 100 μL of 1× Assay Buffer provided with the detection kit.

Then, cells were incubated for 30 min at 37 °C with 100 μL of dual detection reagent (prepared by diluting Cyto-ID^®^ Green Detection Reagent 330 times in a mixture of 1× Assay Buffer), protected from light. Finally, cells were fixed with 2% paraformaldehyde for 20 min and washed three times with the 1× Assay Buffer. Then coverslips were placed on microscope slides using a Fluoromount™ Aqueous Mounting Medium (Sigma Aldrich-Merck). Fluorescent signals were visualized using a Leica TCS SP5 confocal scanning microscope (Leica, Mannheim, Germany) equipped with a HeNe/Ar laser source to allow fluorescence measurements at 488 nm. The cell observations were performed using a Leica Plan 7 Apo X63 oil immersion objective, suited with optics for DIC acquisition. Cells from three independent experiments and three different fields (about 20 cells/field) per experiment were analysed. The fluorescence intensity was analysed with the ImageJ software (Image 1.51j8 version, National Institutes of Health Bethesda, Bethesda, MD, USA), and expressed as percentage increase respect to untreated cells.

### 3.12. Analysis of IGF-1R Localization

HT1080 cells were plated at a density of 5 × 10^4^ cells per well in a 24-well culture plate containing sterilized glass coverslips and grown for 24 h. Next, cells were treated with 1:500 POE dilution for 0.5, 3, 7 and 16 h, fixed with 2% paraformaldehyde for 5 min and permeabilized with ice cold 50% acetone/50% ethanol solution for 4 min at RT. After PBS washing, cells were blocked in saturated solution (0.5% BSA and 2% gelatin) for 30 min at 37 °C. After 1 h of incubation at 37 °C with a mouse anti-IGF-1R monoclonal antibody (Cell Signaling) diluted to 1:100 in saturated solution, cells were washed with PBS for 30 min under stirring conditions and then incubated with Alexa 488-conjugated anti-mouse secondary antibody (Invitrogen Molecular Probes) diluted to 1:200 in PBS for 1 h at 37 °C in the dark. Finally, cells were washed twice with PBS, and coverslips were placed onto microscope slides using a Fluoromount™ Aqueous Mounting Medium. Fluorescent signals were visualized using a Leica TCS SP5 confocal scanning microscope (Leica, Mannheim, Germany) equipped with a HeNe/Ar laser source for fluorescence measurements. The observations were performed using a Leica Plan 7 Apo X63 oil immersion objective, suited with optics for DIC acquisition.

### 3.13. Detection of Autophagy Markers

HT1080 cells (2 × 10^5^ cells) were seeded in 60 mm dishes in HI-FBS medium condition and were incubated for 24 h. Subsequently, cells were treated with 1:500 POE dilution and after PBS washing they were lysed at different time points, ranging from 0.5 h to 24 h, in 150 μL of Laemmli buffer (62.5 mM Tris-HCl pH 6.8, 10% (*w*/*v*) SDS, 25% (*w*/*v*) glycerol) without bromophenol blue. The protein concentration of lysates was measured by a BCA protein assay. Equal amounts of cellular lysates (15 μg), added with β-mercaptoethanol and bromophenol blue, were resolved by 12% PAGE and transferred onto nitrocellulose membranes. After blocking with 5% (*w*/*v*) BSA in 0.1% (*v*/*v*) PBS-Tween-20 for 1 h, membranes were incubated overnight at 4 °C with the primary antibodies of specific protein markers involved in autophagy signaling listed in Table 2. Then, nitrocellulose membranes were washed three times in 0.1% (*v*/*v*) PBS-Tween-20 and were incubated for 1 h with specific goat anti-rabbit (Invitrogen Molecular Probes) and goat anti-mouse secondary antibodies (Invitrogen Molecular Probes) at a dilution of 1:10,000 in blocking buffer. After washing in 0.5% (*v*/*v*) PBS-Tween-20, specific protein bands were detected using Clarity Western ECL solution, and chemiluminescent signals were acquired by using Amersham TM 600 Imager (GE Healthcare Life Science, Pittsburgh, PA, USA) imaging system. Immunoreactive bands were quantified by Quantity One software (4.6.6 version, Bio-Rad).

### 3.14. Data Analysis and Figure Preparation

Where not otherwise specified, data are reported as the mean ± standard error of values from three independent experiments, after mean centering as a normalizing strategy across experiments. Plots were drawn with LibreOffice Calc, and panels were assembled with LibreOffice Impress and further adapted with Gimp 2.8.

## 4. Conclusions

In this work, we evaluated the contribution of autophagy to the previously demonstrated reduction of cell migration after POE treatment [3]. After verifying that the HT1080 cell line, a well-known model of cell migration, exhibits a motility phenotype in the absence of starvation (it was important in order to avoid a basal increase in autophagy), we demonstrated that the effects of POE compounds on motility reduction are highly correlated with a transient autophagy increase that has no detectable effect on cell viability.

Usually, the mechanisms of action of anti-cancer drugs are based essentially on the differential cell toxicity and sensibility of actively growing cancer cells with respect to normal cells. Our results demonstrate the potential of POE bioactive compounds to work against cell invasion (e.g., metastasis) with a completely non-toxic mechanism. *P. oceanica* decoction has been historically used as a vitalizer and traditional remedy for diabetes in Anatolia villages, and the administration of *P. oceanica* extract to rats showed no signs of toxicity [1]. Further studies will be needed to confirm the absence of POE toxicity in humans under experimentally controlled conditions and, more importantly, to establish its anti-metastatic effects in vivo. Nevertheless, this work documents important potential therapeutic properties of *P. oceanica* compounds regarding the prevention of malignancies and other physio-pathological chronic processes, such as neurodegeneration, inflammation and skin aging, in whose progression gelatinolytic activity is the hallmark.

## Figures and Tables

**Table 1:** *P. oceanica* extract biochemical composition. All values are reported as means ± standard deviations from at least three independent extractions and are expressed in mg/mL of extract after resuspension, as described in the text.

	Polyphenols	Antioxidant Activity	Radical Scavenging	Carbohydrates
Method	Folin–Ciocalteau	Ferrozine®	DPPH	Phenol/Sulfuric acid
Reference control	Gallic acid	Ascorbic acid	Ascorbic acid	Glucose
POE	5.7 ± 0.3	1.5 ± 0.3	12.8 ± 0.7	13 ± 2

**Table 2:** Primary antibodies used in Western blotting experiments.

Antibody	Target	Dilution	Host	Source
SQTSM1/p62	SQTSM1/p62 protein	1:1000	Rabbit	Abcam
LC3A/B	Microtubule-associated protein light chain 3 (A/B)	1:1000	Rabbit	Cell Signaling
P-AKT1	P-AKT1 serine/threonine kinase (Ser473)	1:5000	Rabbit	Abcam
AKT1/2	AKT1/2 serine/threonine kinase	1:5000	Rabbit	Abcam
p44/42 MAPK(ERK1/2)	Mitogen-activated protein kinases p44/42 (ERK 1/2)	1:2000	Mouse	Cell Signaling
P-p44/42 MAPK(ERK 1/2)	Mitogen-activated protein kinases p44/42 (ERK 1/2) (Thr202/Thr204)	1:1000	Rabbit	Cell Signaling
Beclin-1	Beclin-1 protein	1:1000	Rabbit	Cell Signaling
S6	Ribosomial protein S6	1:1000	Rabbit	Cell Signaling
P-S6	Ribosomial protein S6 (Ser235/236)	1:2000	Rabbit	Cell Signaling
Alpha-Tubulin	Alpha-Tubulin protein	1:1000	Mouse	Cell Signaling
Actin	Actin protein	1:1000	Mouse	Santa Cruz
